# Nutritional Care in Patients with Head and Neck Cancer during Chemoradiotherapy (CRT) and Bioradiotherapy (BRT) Provides Better Compliance with the Treatment Plan

**DOI:** 10.3390/cancers13112532

**Published:** 2021-05-21

**Authors:** Aleksandra Kapała, Agnieszka Surwiłło-Snarska, Magdalena Jodkiewicz, Andrzej Kawecki

**Affiliations:** 1 Department of Clinical Nutrition, Head and Neck Cancer Department, Maria Sklodowska-Curie National Research Institute of Oncology, 02781 Warsaw, Poland; 2 Department of Clinical Nutrition, Maria Sklodowska-Curie National Research Institute of Oncology, 02781 Warsaw, Poland; agnieszka.surwillo@pib-nio.pl (A.S.-S.); magdalena.jodkiewicz@pib-nio.pl (M.J.); 3 Head and Neck Cancer Department, Maria Sklodowska-Curie National Research Institute of Oncology, 02781 Warsaw, Poland; andrzej.kawecki@pib-nio.pl

**Keywords:** malnutrition, head and neck cancer, chemoradiotherapy, bioradiotherapy, clinical nutrition

## Abstract

**Simple Summary:**

The nutritional status of patients with head and neck cancer (HNC) during concurrent chemoradiotherapy (CRT) with platinum derivatives or concurrent radiotherapy with cetuximab (bioradiotherapy, BRT) inevitably deteriorates during treatment. Malnutrition is responsible for increased treatment-related toxicity, an increased incidence of infectious complications, the risk of postponing or discontinuing therapy, reduced drug doses (platinum derivatives or cetuximab), deteriorating quality of life (QoL), worse outcome, and increased treatment costs. A nutritional care programme, which included prophylactic dietary counselling and early enteral nutrition, reduced the incidence of complications and prevented drug dose reduction and the deterioration of patients’ anthropometric and laboratory parameters. The study confirmed that nutritional care before and during CRT and BRT in patients with HNC is a determinant of therapeutic benefit.

**Abstract:**

The treatment of locally advanced head and neck cancer (HNC) is based on extensive resections followed by concurrent chemoradiotherapy (CRT) with platinum derivatives or concurrent radiotherapy with cetuximab (bioradiotherapy; BRT). Malnutrition, which occurs in up to 60% of patients before treatment commencement, severely increases the risk of CRT/BRT drug dose reductions and the incidence of treatment-related adverse events. A prospective observational study was performed regarding the influence of nutritional care on nutritional status, compliance with the treatment’s planned regimen, and the incidence of treatment-related complications in patients with advanced HNC during CRT and BRT. The study population encompassed 153 patients compared with a retrospective control group of 72 patients treated before nutritional care was included in the standard of oncological care. Patients enrolled in the nutritional care programme received significantly higher doses of platinum derivatives or cetuximab than patients in the control group. A significant difference between the compared populations was observed in patients below 70 years of age (92.8% of the study population), after prior surgery, and with initial weight loss lower than 10%. Nutritional care reduced final weight loss and prevented a decline within the laboratory markers of nutritional status. Weight loss was comparable in both modes of treatment—CRT and BRT. The incidence of treatment-related complications was significantly higher in patients without nutritional support in the subgroups of patients under 70 years of age and after primary surgery. Nutritional care before and during CRT and BRT in patients with HNC is a determinant of therapeutic benefit, defined as preventing down-dosing, weight loss, and the incidence of complications. Platinum derivatives and cetuximab had comparable influence on weight loss.

## 1. Introduction

Head and neck cancer (HNC) is responsible for approximately 330,000 deaths yearly [1]. Early clinical stages (I–II, cT1–2. N0) can be permanently cured in 60% to 90% by means of surgery, radiotherapy (RTH), or both [2]. In clinical practice, treatment of patients with locally advanced head and neck squamous cell carcinoma poses a challenge because of tumour-related, patient-related, and treatment-related factors [3,4].

However, many patients disease recurs; the recurrence rate in early stage HNC is ≈10–20% [5], whereas the recurrence rate in locally advanced HNC (CS III–IV, cT3–T4, and/or N1–N3) is ≈50% with a predominance of locoregional failure [6,7,8]. Patients with recurrent or metastatic (R/M) SCCHN have a poor prognosis with median overall survival (OS) of under 1 year [9].

Therefore, the treatment is based on extensive resections followed by concurrent chemoradiotherapy (CRT) with platinum derivatives or concurrent RTH with cetuximab (BRT) or on definitive concurrent chemoradiotherapy in inoperable cases [10,11].

Generally, the benefit of therapy is dependent on a commitment to the planned regimen. Treatment interruptions prolong the overall treatment time and cause loss of control over cancer cells, thus worsening the treatment’s cytoreductive effect. A decrease in treatment intensity worsens the objective response rate and long-term survival and is observed after both RTH and chemotherapy de-escalation [12]. Receiving no less than 50 Gy of cumulative RTH dose confers a therapeutic benefit [13,14]. Higher cumulative doses of weekly cisplatin regimens have superior efficacy in progression-free survival and overall survival compared to regimens with lower weekly doses [15]. The recommended cumulative dose of concomitant cisplatin used along with RTH is over 200 mg/m^2^ [14,15]. A decrease in the cytostatic dose even by 20% worsens therapeutical outcomes by approximately 50% [16]. Therefore, the dose reductions in patients with HNC that occur even in 41–61% are highly disadvantageous [14,17].

Alternatively, CRT is an aggressive treatment method that is greatly limited by adverse events in more than 50% of patients. The most common complications include mucosal and skin radiation reactions, dysphagia, weight loss, pain, nephrotoxicity, neurotoxicity, ototoxicity, myelosuppression, increased risk of infections, and treatment interruption and discontinuation. In recent years, BRT with cetuximab has gained a good reputation as being safer than platinum derivatives [18] because it results in less organ toxicity [19,20]. However, in patients with HNC, the most severe factors in the incidence and severity of adverse events are weight loss and malnutrition, which develop in up to 60% of patients before treatment and progresses to 75–95% regardless of the treatment method [21,22,23]. Malnutrition increases the risk of treatment discontinuation, attenuates the response to treatment, prolongs hospitalisation, worsens disease-free survival, increases mortality, and dramatically reduces performance status and quality of life [24,25,26,27,28,29,30,31,32,33]. Thus far, there are no data comparing platinum and cetuximab in terms of the development and progression of malnutrition.

## 2. Materials and Methods

The patients in the prospective observational study were compared to the retrospective control group in regard to the benefits of nutritional care in HNC patients treated with CRT or BRT. The benefit was defined as a commitment to a treatment plan. Study endpoints were weight loss during treatment, the used percentage of the planned dose of platinum derivatives or cetuximab, deterioration in anthropometric and laboratory markers of nutritional status, and the incidence of treatment-related complications. The analyses included additional factors related to malnutrition, such as age, previous surgery, and initial weight loss. The study was performed in the Head and Neck Cancer Department in the Cancer Centre-Maria Sklodowska-Curie Institute in Warsaw, Poland and received the favourable opinion of the Bioethical Commission (KB 078-9/18).

### 2.1. Population and Treatment Regimen

The study population included 153 patients treated with CRT or BRT for the radical treatment of primary squamous cell carcinoma of the head and neck and enrolled in a nutritional care programme. Patients were recruited consecutively from July 2016 to April 2018. A total of 91.5% of patients were treated with CRT and 8.5% with BRT. Cisplatin in the dose of 100 mg/m^2^ every three weeks was used in 137 cases, and carboplatin (area under the curve (AUC) 5 mg/mL/min) was used in only four cases. The cetuximab regimen was started at 400 mg/m^2^, with subsequent 250 mg/m^2^ every seven days. The control population consisted of 72 patients who had undergone CRT with cisplatin between 2010 and 2012. At that time, cetuximab was unavailable in Poland, and nutritional care with dietary counselling was not included in the standard of care. The most frequently used irradiation technique was intensity modulated RTH (IMRT) with a standard cumulative dose of 70–72 Gy administered for six to seven weeks in fractions of 1.8–2.0 Gy per day. In both populations, some patients had previously undergone radical surgery, and some received neoadjuvant chemotherapy. In both populations, oropharyngeal and laryngeal cancers were most frequently diagnosed. Cancers of the floor of the mouth and other types of cancer, including cancers of the lips, nasal cavity, and nasal sinuses, were the least common. Cancer of unknown origin was found in 5% of cases in each population. The stage of local and nodal advancement of the tumours met the criteria for CRT treatment. None of the patients had metastases to distant locations. The incidence of PEG and TF (tube feeding) were comparable in both groups. Detailed data on the study and control population are presented in Table 1.

### 2.2. Nutritional Care Programme

The nutritional management of a patient with HNC qualified for CRT/BRT was defined as a nutritional care programme. It aimed to carefully monitor the general and nutritional status of patients and to implement adequate nutrition throughout the treatment period. In line with the scientific suggestions for the benefits of prophylactic placement of a percutaneous endoscopic gastrostomy (PEG) in preventing of CRT-related malnutrition [33,34,35], each patient in the study group was proposed the procedure before beginning CRT or BRT. The procedure was performed only in 31.4% of patients (*n* = 48) due to a lack of consent from the remaining patients. At the beginning of concurrent treatment, each patient underwent a medical qualification with an evaluation of the level of blood counts, electrolytes, creatine and GFR, glucose, albumins, CRP, and markers of liver function as well as an anthropometric assessment including body weight and height measurements, BMI calculation, initial weight loss assessment, NRS 2002 scale score, and calculation of daily energy demand (kcal). Regular control of these parameters and an ongoing evaluation of swallowing abilities, the effects of treatment and the incidence of CRT/BRT-related toxicities were repeated every 14 days at follow-up visits until the end of treatment and hospital discharge.

**Figure 1:**
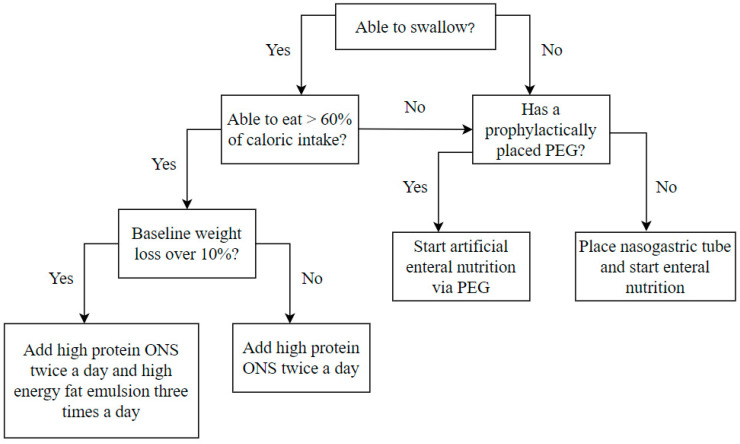
Nutrition algorithm included in the nutritional care program performed at the beginning and every 14 days during the treatment period. ONS oral nutritional supplements, PEG – percutaneous endoscopic gastrostomy.

Regardless of the initial nutritional status at the beginning of the treatment, all patients were supplemented with complete high-protein oral nutritional supplements (ONS), which provided an additional 600–800 calories per day, including 36–38 g of protein. In the case of initial weight loss, over 10% of patients were additionally supplemented with a high-energy fat emulsion that provided an additional 400 calories daily. A significant decrease in oral intake abilities, defined as the reduction of oral intake to less than 60% of daily energy demand, typically occurred in the fourth week of treatment. Then, artificial enteral nutrition was introduced through the previously placed PEG or a nasogastric tube placed according to clinical indications. The high-protein and high-energy artificial enteral diet provided 30–35 calories per kilogram of body weight per day. After the end of CRT/BRT, enteral nutrition was continued as home enteral nutrition until adequate oral food intake was restored. The nutrition algorithm is presented in Figure 1.

The nutrition of patients in the control group during CRT was not supported by dietary counselling and did not differ from the typical hospital diet (hospital catering). In severe dysphagia, patients were fed enterally via PEG or nasogastric tube with a hospital mixed diet. Only seven patients were fed with an artificial enteral diet, but retrospective calculations showed that their intake was below their energy requirements.

### 2.3. Statistics

The normal distribution of variables was investigated and confirmed by the Kolmogorov–Smirnov test. Group comparisons were performed using non-parametric tests due to the different numbers of patients in the study and control populations. The Mann–Whitney test was used to compare the percentage of the planned dose of the drug and the loss of body weight in the study and control populations. The assessment of the variability of the percentage of drug used and weight loss in the study and control populations in terms of additional factors affecting the final weight loss was performed using two-way analyses of variance for independent trials in a mixed 2 × 2 scheme. The influence of age and primary surgery on the incidence of complications in the control and study groups was analysed using the chi-squared test or Fisher’s exact test, depending on the size of the compared groups. Statistics were performed using programme made by IBM Corp. released 2017, IBM SPSS Statistics for Windows, Version 25.0, Armonk, NY: IBM Corp.; *p* = 0.05 was used statistically.

## 3. Results

### 3.1. Influence of Nutritional Care on the Weight Loss and Deterioration of Nutritional Status Markers during Therapy

Patients in the control population who were not adequately fed presented significantly higher weight loss and higher decline within the level of white blood cells, neutrophils, and albumin concentration during therapy than the study population (Table 2 and Table 3IA).

**Figure 2:**
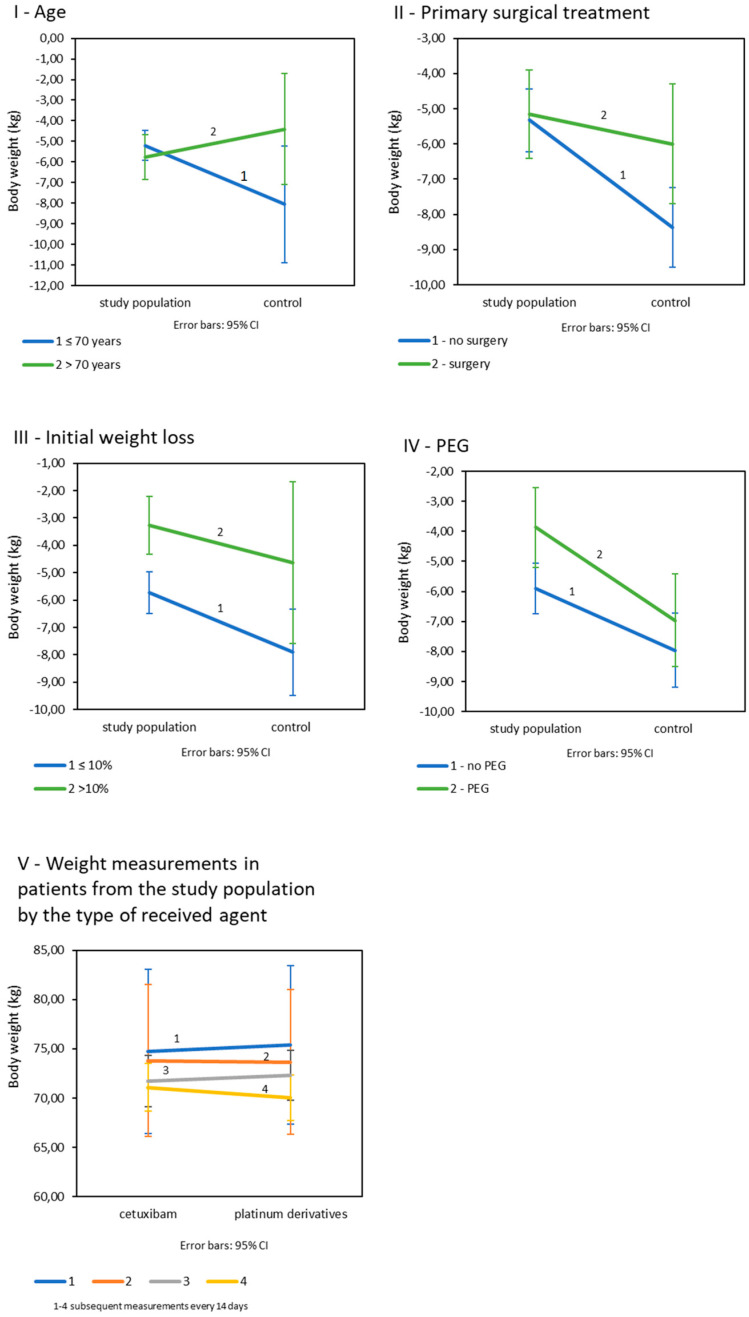
Mean weight loss in study and control populations depending on: (**I**): age; (**II**): primary surgical treatment; (**III**): initial weight loss; (**IV**): prophylactic PEG placement (**V**): mean weight loss in control measurements in patients from the study population by the type of received drug (*n* = 225).

**Age:** Age was shown to significantly affect weight loss in all patients (*p* = 0.045). Significant differences in the final weight loss between the subject and the control groups occurred in patients under the age of 70 as opposed to older patients (Table 3IB; Figure 2I). There were no significant differences in age-related weight loss in the study group (*p* = 0.709), but in the control population, weight loss was significantly greater in patients under 70 years of age and was on average −8.05 kg (SD = 5.61. *p* = 0.014). **Primary surgical treatment:** The final weight loss appeared to be dependent on the history of the primary surgery. There was a trend of greater weight loss in patients in both cohorts after surgery, and the statistics were close to significance (*p* = 0.050) (Table 3IC; Figure 2II). **Initial weight loss:** Differences in final weight loss between the compared populations occurred in patients whose initial weight loss was less than 10%. Such a relationship was not observed in patients with an initial weight loss greater than 10% (Table 3ID; Figure 2III). In both the study and control populations, patients with initially less weight loss eventually lost significantly more kilograms than patients with greater initial weight loss (*p* = 0.006 for the study population and *p* = 0.040 for the control group). **Prophylactic PEG placement:** Patients in study group fed with PEG lost significantly less kilograms (M = −3.87; SD = 3.0) than patients not fed with PEG (M = −5.91; SD = 3.80); *p* = 0.008. In the control group, weight loss by PEG was statistically insignificant (*p* = 0.337); (Table 3IE; Figure 2IV). All interaction effects for tested variables are summarized in supplementary materials (Table S1).

In the study population, the final weight loss in patients treated with platinum derivatives and cetuximab was not significantly different (*p* = 0.127). Body weight decreased significantly in every 14-day control measurement during both CRT and BRT (*p* < 0.001), and there were no differences between patients treated with platinum derivatives and cetuximab (Figure 2V).

### 3.2. Influence of Nutritional Care on the Doses of Drugs Used during CRT/BRT

**Figure 3:**
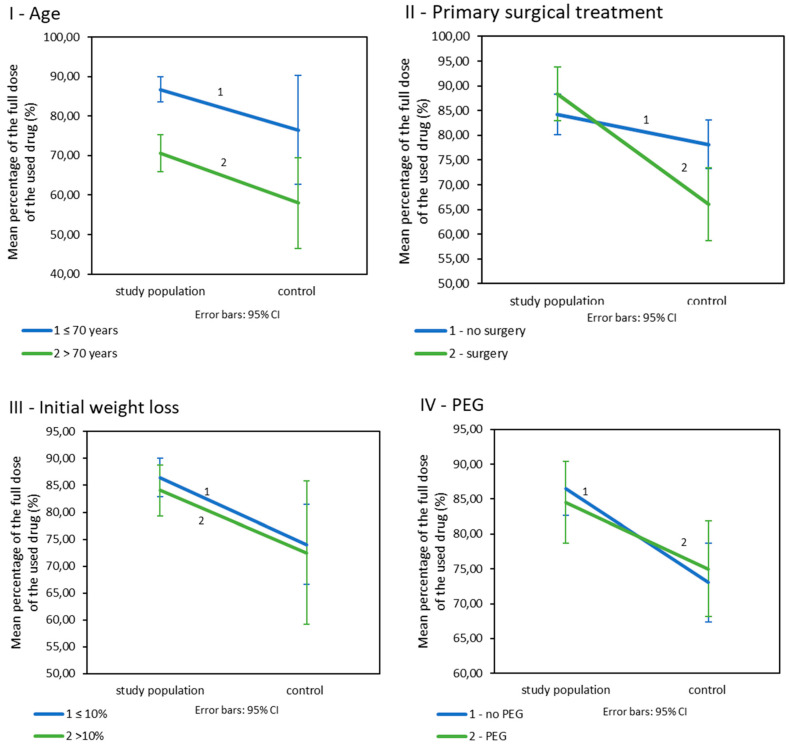
The percentage of the drug’s planned dose used in CRT/BRT in the study and control population by (**I**): age. (**II**): primary surgical treatment. (**III**): initial weight loss. (**IV**): prophylactic PEG placement, (*n* = 225).

Patients included in the nutritional care programme were treated with significantly higher doses of drugs than patients in the control group (Table 3IIA). Age: In patients below 70 years of age, the study population had fewer reduced doses of drugs than their counterparts from the control population (Table 3IIB; Figure 3I). The dose levels in both populations were dependent on age. Younger patients were treated with higher doses than older patients (*p* = 0.026 for the study population and *p* = 0.004 for the control population). Primary surgical treatment: Patients enrolled in the nutritional care programme who underwent primary surgery were treated with significantly higher doses of drugs than their control group matches. Such a difference was not observed in patients without surgery (Table 3IIC; Figure 3II). Additionally, in the study population, the used doses of drugs did not significantly vary depending on the presence or absence of surgery (*p* = 0.199) but did in the control population (*p* = 0.009). Initial weight loss: Initial weight loss did not affect drug doses (*p* < 0.001). However, patients in the study population with an initial weight loss of less than 10% received significantly higher doses of drugs than patients from the control group. Such a difference was not observed in patients who lost more than 10% of their initial weight loss before treatment (Table 3IID; Figure 3III). Prophylactic PEG placement: Patients in the study group received higher doses of drugs in combination with radiotherapy; however, this was not related to the presence of PEG. The differences between the study groups are small and statistically insignificant *p* > 0.005 (Table 3IIE, Figure 3IV).

### 3.3. Influence of Nutrition Care on the Rate of Adverse Events during CRT

Adverse events were reported in both populations, in 16.6% of the population included in the nutritional care programme and 25.4% of the control group of patients without nutritional support. Infectious complications occurred in 11.1% of patients in the study group and 20.8% in the control group. Pneumonia and infectious exacerbations of chronic obstructive pulmonary disease were the most common. The non-infectious complications included deterioration of renal and hepatic function, thrombocytopenia, and a case of severe depression. Age: In patients under 70 years of age, the incidence of adverse events was significantly higher in the control group than in the study population (Table 3IIIB). Primary surgical treatment: In addition, control patients who had previously undergone surgery had more complications than patients in the study group (Table 3IIIC). Prophylactic PEG placement: PEG presence does not affect incidence of adverse events *p* > 0.005 (Table 3IIIE).

## 4. Discussion

The presented study results are of practical importance in the treatment of patients with advanced HNC, who constitute a specific population of patients. Clinical experience confirms that HNC treatment may be discontinued due to the often poor CRT tolerance, and completing the whole treatment plan can be compromised. Mollnar showed that in a study of 193 patients, the RTH dose was reduced in 6% of patients and CTH in 64% [12]. In our study, CRT or BRT modifications occurred in 49.6% in the study group and in 83% of patients in the control retrospective population, respectively. Frequent systemic treatment failures appear to be highly disadvantageous as concurrent systemic therapy sensitises cancer cells to RTH and improves long-term survival rates by 7–25% [36]. Compulsory reduction in the intensity of therapy is often caused by progressive malnutrition and the initially poor condition of many patients caused by addiction to tobacco and alcohol and low socio-economic status [37]. It had already been shown that better-nourished patients have a better tolerance of treatment-related toxicities, a greater chance of recovery, a lower rate of complications, better compliance, and, in consequence, receive a greater benefit of anticancer treatment [32,38,39,40]. The performed analyzes also show the importance of prophylactic PEG placement. In the study group, weight loss was significantly lower if the patients were fed via PEG. Such dependencies were not found in the control group. In the study group, patients received only industrial enteral diets via PEG, while in the historical group, a home mixed diet was provided. This confirms that not only the type of access to the gastrointestinal tract is important, but also the type of diet, its composition, and an adequate amount under the supervision of a dietitian [41]. An important result of our study is a significant difference in the clinical benefit of patients under the age of 70 between the compared populations. In this age group, which represented 92.8% of the study population, patients who were enrolled in the nutritional care programme had significantly lower final weight loss, were treated with higher doses of drugs, and had fewer treatment related-toxicities, especially infectious complications, than the control group. Given previous data that patients under 70 years of age benefit most from the concomitant chemotherapy and that this benefit decreases with age [36], our results confirm that nutritional care is an indirect means of improving treatment effects.

We expected that nutritional support would affect the incidence of complications regardless of age. However, the observed lack of significant differences between the compared populations of patients over 70 could result from the small size of this age group and a significantly reduced treatment regimen. The percentage of patients over 70 years of age in the study and control populations was 7.2% and 15.3%, respectively. They were treated with only about 70% and 58% of the planned dose of the drug, respectively. The oldest population was also a selected group of patients with good performance status and minor comorbidities due to the highest risk of RTH-related toxicity [40,42].

Another clinically relevant study finding was the observation that primary surgical treatment of patients enrolled in the nutritional care programme was a factor of a lower incidence of complications and better compliance with the systemic therapy, that is, a higher percentage of the planned drug’s dosage than in the control group. Surgery is a big challenge for the patient’s general condition and is an independent factor in the development of complications, as has already been demonstrated in other studies [18,31]. Radical surgeries for HNCs are rather extensive resections and are often combined with simultaneous reconstruction or adjuvant RTH with or without systemic treatment, which altogether impairs wound healing. In such cases, lack of nutritional support dramatically increases the complication rate [18,31,43]. In our study, no significant differences in the intensity of drug dosage were found with and without primary surgery in patients under nutritional treatment, which confirmed that careful nutrition improves recovery after surgery and increases tolerance to adjuvant therapy. Thus, we showed that a simple means such as nutrition support allows one to easily achieve a significant clinical benefit.

Another clinically relevant finding of the study was the comparable effect of platinum derivatives and cetuximab on weight loss during CRT and BRT. Maintaining proper weight in HNC patients positively influences the long-term outcomes of the treatment. None of the treatment regimens in our study prevented weight loss. Our finding is supported by similar data [20,44], which confirmed that toxicity of platinum derivates and cetuximab is comparable. However, other authors have shown that BRT with cetuximab resulted in poorer overall survival, progression-free survival, locoregional control, and distant metastasis-free survival than cisplatin-based therapy [11,20,45]. Altogether, these results practically reduce the value of cetuximab in the treatment of advanced HNC, but cetuximab still remains a valuable option for patients with contraindications to platinum-based therapy.

## 5. Conclusions

The study confirmed that nutritional care before and during CRT and BRT in patients with HNC is a determinant of therapeutic benefit. The most significant benefits of nutritional care were seen in patients under 70 and after primary surgery. In these patients, statistically lower drug dose reductions and lower incidence of complications were observed in patients with nutritional support. Additionally, weight loss was significantly lower in the younger patients enrolled in the nutritional care programme. Platinum derivatives and cetuximab had comparable and negative effects on weight loss.

## Figures and Tables

**Table 1:** Characteristics of populations (*n* = 225). BMI: body mass index; PEG: percutaneous endoscopic gastrostomy, TF: tube feeding, CTH: chemotherapy, CRT: chemoradiotherapy.

Variable	Study Population	Control Population	*p*
**Total number of patients**	153	72	
**Sex**			
Male	79.8% (122)	86.1% (62)	0.248
Female	20.2% (31)	13.9% (10)	
**Mean age** (years)	58	61.7	0.007
≤70 years old	92.8% (142)	84.7% (61)	
>70 years old	7.2% (11)	15.3% (11)	
**BMI M (SD)**	25.60 (4.32)	26.23 (5.24)	0.341
**PEG**	31.4% (48)	41.7% (30)	0.130
**TF**	17.2% (26)	21.1% (15)	0.609
**Undergone surgery**			
Yes	37.9% (58)	34.7% (25)	0.644
No	62.1% (95)	65.3% (47)	
**Neoadjuvant CTH**			
Yes	28.8% (41)	18.1% (13)	0.152
No	71.2% (112)	81.9% (59)	
**The agent used in CRT**			
Cisplatin/Carboplatin	91.4% (140)	100% (72)	0.011
Cetuximab	8.6% (13)	0% (0)	
**Tumor site**			
Nasopharynx	11.1% (17)	9.7% (7)	0.933
Oropharynx	30.1% (46)	37.5% (27)	0.338
Laryngopharynx	13.1% (20)	2.8% (2)	0.029
Tongue	14.4% (23)	8.3% (6)	0.236
Floor of the mouth	3.9% (6)	1.4% (1)	0.434
Larynx	18.3% (28)	31.9% (23)	0.035
Other	3.9% (6)	2.8% (2)	1.000
Unknown	4.6% (7)	5.6% (4)	0.748
**Tumor**			
Tx	5.2% (8)	5.6% (4)	1.000
T1	15.7% (25)	9.7% (7)	0.262
T2	19.0% (29)	30.6% (22)	0.077
T3	28.0% (43)	27.8% (20)	1.000
T4	31.4% (48)	26.4% (19)	0.544
**Nodules**			
Nx	1.3% (2)	1.4% (1)	1.000
N0	9.8% (15)	8.3% (6)	0.914
N1	21.6% (34)	19.4% (14)	0.764
N2a	11.8% (18)	11.1% (8)	1.000
N2b	18.3% (28)	33.3% (24)	0.020
N2c	23.5% (36)	18.1% (13)	0.450
N3	13.1% (20)	8.3% (6)	0.416

**Table 2:** Levels of laboratory markers of nutritional status after CRT/BRT (*n* = 225).

Variable	Study Population		Control		Statistical Significance
WBC (G/L)	7.66	SD = 8.08	4.75	SD = 4.26	*p* < 0.001
NEUT (G/L)	6.28	SD =7.78	3.4	SD = 3.83	*p* < 0.001
HGB (G/dL)	11.91	SD = 1.47	12.01	SD = 1.48	*p* = 0.631
ALB (G/L)	37.77	SD = 3.68	33.86	SD = 4.57	*p* < 0.001

WBC: White blood cells; NEUT: Neutrophils; HGB: Hemoglobin; ALB: Albumin.

**Table 3:** Final weight loss (**I**), used the percentage of the planned dose of drugs (**II**) and incidence of adverse events (**III**) in study and control populations in general (**A**) or depending on factors that worsen prognosis, i.e., age (**B**), primary surgical treatment (**C**), initial weight loss (**D**), prophylactic PEG placement (**E**); (*n* = 225).

Variable	I Weight Loss (kg)			II Used Percentage of the Planned Dose of Drugs (%)			III The Incidence of Adverse Events (%)		
	Study	Control		Study	Control		Study	Control	
**A General**									
	−5.26 SD = 3.68	−7.54 SD = 5.46	***p* = 0.006**	85.90 SD = 19.28	73.87 SD = 18.33	***p* < 0.001**	16.3	25	*p* = 0.123
**B Depending on the age**									
≤70 years old	−5.20 SD = 3.70	−8.05 SD = 5.61	***p* < 0.001**	86.69 SD = 19.13	76.48 SD = 16.54	***p* < 0.001**	14.9	27.9	***p* = 0.03**
>70 years old	−5.76 SD = 3.52	−4.40 SD = 3.17	*p* = 0.493	70.57 SD = 18.09	58.0 SD = 21.49	*p* = 0.493	33.3	10	*p* = 0.30
**C Depending on primary surgical treatment**									
Surgery	−5.15 SD = 3.57	−6.00 SD = 5.40	*p* = 0.414	88.38 SD = 17.86	66.00 SD = 20.92	***p* < 0.001**	7	28	***p* = 0.029**
No surgery	−5.33 SD = 3.77	−8.37 SD = 5.37	*p* < 0.001	84.21 SD = 20.13	78.15 SD = 15.36	*p* = 0.080	22.3	23.9	*p* = 0.83
**D Depending on initial weight loss**									
≤10%	−5.73 SD = 3.71	−7.91 SD = 5.51	***p* = 0.001**	86.43 SD = 18.96	74.05 SD = 18.81	***p* < 0.001**	**17.2**	25.4	*p* = 0.187
>10%	−3.26 SD = 2.85	−4.63 SD = 4.31	*p* = 0.425	84.04 SD = 21.12	72.50 SD = 14.88	*p* = 0.136	14.3	25.0	*p* = 0.597
**E Depending on PEG**									
No	−5.91 SD = 3.80	−7.96 SD = 4.94	***p* = 0.030**	86.53 SD = 19.16	73.05 SD = 18.16	*p* = 0.065	17.1	28.6	*p* = 0.120
Yes	−3.87 SD = 3.00	−6.97 SD = 6.16	***p* = 0.043**	84.55 SD = 19.70	75.00 SD = 18.80	***p* = 0.035**	14.6	20.0	*p* = 0.532

*p*-value in the table = *p*-value of post hoc analysis (for ANOVA—*p*-value for simple effects); Bold-statistically significant.

## Data Availability

The data presented in this study are available on request from the corresponding author. The data are not publicly available due to privacy and ethical issue.

## Supplementary Materials

The following are available online at https://www.mdpi.com/article/10.3390/cancers13112532/s1, Table S1: The interaction effect for age, primary surgical treatment, initial weight loss, PEG.
