# Supplementary material for: Nutritional Care in Patients with Head and Neck Cancer during Chemoradiotherapy (CRT) and Bioradiotherapy (BRT) Provides Better Compliance with the Treatment Plan
Source: Cancers (Basel). 2021 May 21;13(11):2532. doi: 10.3390/cancers13112532 (PMC8196687; doi:10.3390/cancers13112532)
Supplement: Supplementary file 1 [file cancers-13-02532-s001.zip › cancers-1181044-supplementary.pdf]

cancers

MDPI

Supplementary Materials: Nutritional Care in Patients with Head and Neck Cancer During Chemoradiotherapy (CRT) and Bioradiotherapy (BRT) Pro-Vides Better Compliance with the Treatment Plan

Aleksandra Kapała, Agnieszka Surwiłło-Snarska, Magdalena Jodkiewicz and Andrzej Kawecki

Table S1. The interaction effects for age, primary surgical treatment, initial weight loss, PEG.

Interaction Effects	Weight Loss	Used Percentage of the Planned Dose of Drugs (%)
Age	$F(1214) = 2{,}22$; $p = 0{,}138$; $\eta^2p = 0{,}010$	$F(1204) = 13{,}11$; $p < 0{,}001$; $\eta^2p = 0{,}060$
Group	$F(1214) = 0{,}51$; $p = 0{,}474$; $\eta^2p = 0{,}002$	$F(1204) = 5{,}69$; $p = 0{,}018$; $\eta^2p = 0{,}027$
Age × Group	$F(1214) = 4{,}08$; $p = 0{,}045$; $\eta^2p = 0{,}019$	$F(1204) = 0{,}06$; $p = 0{,}806$; $\eta^2p < 0{,}001$
Primary surgical treatment	$F(1215) = 3{,}88$; $p = 0{,}050$; $\eta^2p = 0{,}018$	$F(1205) = 8{,}32$; $p = 0{,}004$; $\eta^2p = 0{,}039$
Group	$F(1215) = 9{,}02$; $p = 0{,}003$; $\eta^2p = 0{,}040$	$F(1205) = 25{,}26$; $p < 0{,}001$; $\eta^2p = 0{,}110$
Primary surgical treatment × Group	$F(1215) = 2{,}88$; $p = 0{,}091$; $\eta^2p = 0{,}013$	$F(1205) = 1{,}99$; $p = 0{,}160$; $\eta^2p = 0{,}010$
Initial weight loss	$F(1215) = 9{,}92$; $p = 0{,}002$; $\eta^2p = 0{,}044$	$F(1204) = 0{,}23$; $p = 0{,}634$; $\eta^2p = 0{,}001$
Group	$F(1215) = 3{,}76$; $p = 0{,}056$; $\eta^2p = 0{,}017$	$F(1204) = 8{,}36$; $p = 0{,}004$; $\eta^2p = 0{,}039$
Initial weight loss × Group	$F(1215) = 0{,}20$; $p = 0{,}654$; $\eta^2p = 0{,}001$	$F(1204) = 0{,}01$; $p = 0{,}919$; $\eta^2p < 0{,}001$
PEG	$F(1215) = 5{,}62$; $p = 0{,}019$; $\eta^2p = 0{,}070$	$F(1205) < 0{,}01$; $p = 0{,}995$; $\eta^2p < 0{,}001$
Group	$F(1215) = 16{,}23$; $p < 0{,}001$; $\eta^2p = 0{,}636$	$F(1205) = 16{,}07$; $p < 0{,}001$; $\eta^2p = 0{,}073$
PEG × Group	$F(1215) = 0{,}67$; $p = 0{,}414$; $\eta^2p = 0{,}025$	$F(1205) = 0{,}47$; $p = 0{,}494$; $\eta^2p = 0{,}002$
